# 3D bioprinted mesenchymal stem cell laden scaffold enhances subcutaneous vascularization for delivery of cell therapy

**DOI:** 10.1007/s10544-024-00713-2

**Published:** 2024-06-18

**Authors:** Tommaso Bo, Elia Pascucci, Simone Capuani, Jocelyn Nikita Campa-Carranza, Letizia Franco, Marco Farina, Jacopo Secco, Sara Becchi, Rosanna Cavazzana, Ashley L. Joubert, Nathanael Hernandez, Corrine Ying Xuan Chua, Alessandro Grattoni

**Affiliations:** 1 Department of Nanomedicine, Houston Methodist Research Institute, 6670 Bertner Avenue, Houston, TX77030, R8-111 USA; 2 Department of Applied Science and Technology, Politecnico Di Torino, Turin, Italy; 3 School of Medicine and Health Sciences, Tecnologico de Monterrey, Monterrey, NL Mexico; 4 Department of Electronics and Telecommunications, Politecnico Di Torino, Turin, Italy; 5 Department of Surgery, Houston Methodist Hospital, Houston, TX, USA; 6 Department of Radiation Oncology, Houston Methodist Hospital, Houston, TX USA; 7 Department of Medicine, Weill Cornell Medical College, New York, NY, USA

**Keywords:** Regenerative medicine, Tissue engineering, 3D bioprinting, Vascularized scaffold, Mesenchymal stem cells, Cell therapy

## Abstract

Subcutaneous delivery of cell therapy is an appealing minimally-invasive strategy for the treatment of various diseases. However, the subdermal site is poorly vascularized making it inadequate for supporting engraftment, viability, and function of exogenous cells. In this study, we developed a 3D bioprinted scaffold composed of alginate/gelatin (Alg/Gel) embedded with mesenchymal stem cells (MSCs) to enhance vascularization and tissue ingrowth in a subcutaneous microenvironment. We identified bio-ink crosslinking conditions that optimally recapitulated the mechanical properties of subcutaneous tissue. We achieved controlled degradation of the Alg/Gel scaffold synchronous with host tissue ingrowth and remodeling. Further, in a rat model, the Alg/Gel scaffold was superior to MSC-embedded Pluronic hydrogel in supporting tissue development and vascularization of a subcutaneous site. While the scaffold alone promoted vascular tissue formation, the inclusion of MSCs in the bio-ink further enhanced angiogenesis. Our findings highlight the use of simple cell-laden degradable bioprinted structures to generate a supportive microenvironment for cell delivery.

**Graphical Abstract:**

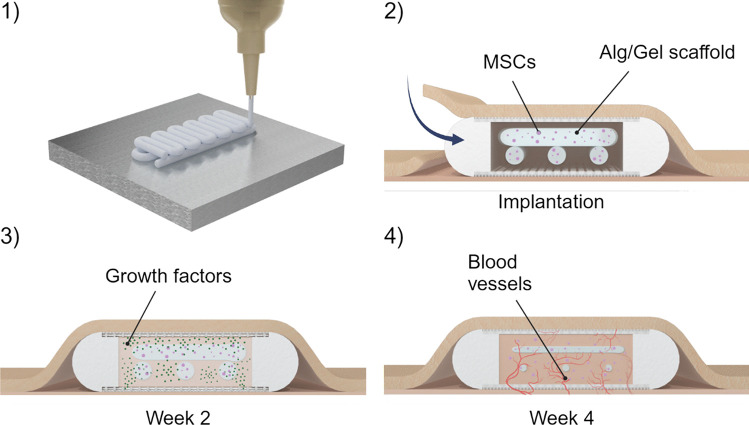

**Supplementary Information:**

The online version contains supplementary material available at 10.1007/s10544-024-00713-2.

## Introduction

Cell therapeutics represent a promising approach for the prevention and treatment of various medical conditions (Murphy and Atala 2014; Pan et al. 2024; Wang et al. 2024). The effectiveness of using autologous and allogeneic cells to restore physiological functions affected by disease is demonstrated in the treatment of numerous pathologies. These conditions include spinal cord injuries, type 1 diabetes, Parkinson's disease, Alzheimer's disease, burns, stroke, cancer, and osteoarthritis, among others (Kim and de Vellis 2009; Lindvall and Björklund 2004; Segers and Lee 2008). However, the successful application of cell therapeutics requires a suitable deployment strategy. Specifically, cells require delivery in a microenvironment that supports their viability, growth, long-term function, and integration within the host tissues. Further, cell delivery would ideally occur within a three-dimensional (3D) space that closely mimics their natural microenvironment (Kim et al. 2019; Ouyang et al. 2020). Moreover, for their viability, cells must have direct access to essential oxygen and nutrients and effectively eliminate metabolic waste. Therefore, to ensure these optimal conditions, a suitable space for their delivery should be well-vascularized, facilitating adequate oxygen and nutrients supply (Rouwkema et al. 2009).

The subcutaneous space is an attractive site for the administration of therapeutic cells due to its accessibility via minimally invasive procedures. This site allows ease of manipulation, supplementation, and retrieval of cells, rendering it a clinically viable choice for cell therapy interventions. However, in contrast to other sites, such as the intraperitoneal cavity and omental bursa, the subcutaneous tissue has limited vasculature (Yu et al. 2020). This presents a challenge, especially when deploying cells with high metabolic demand and oxygen consumption. For example, pancreatic islet transplanted subcutaneously in rats and mice without prior vascularization progressively lose their viability and function (Barkai et al. 2016; Smink et al. 2024).

To this end, several strategies are explored to enhance vascularization in the subcutaneous microenvironment (Pepper et al. 2015). Among these, local supply of pro-angiogenic factors like vascular endothelial growth factor (VEGF) and fibroblast growth factor (FGF) can effectively stimulate the formation of new blood vessels (Quizon et al. 2024; Xu et al. 2018). Similarly, platelet rich plasma (PRP) hydrogels are shown to improve vascularization in various preclinical models (Paez-Mayorga et al. 2020a). Moreover, various materials are evaluated for supporting sustained localization of pro-angiogenic factors including vascularizing degradable methacrylic acid-polyethylene glycol (MAA-PEG) and PEG hydrogels (Kinney et al. 2022; Weaver et al. 2017), hydroxypropylmethyl cellulose hydrogel integrated with plasma components (Schaschkow et al. 2020), collagen, and glutamine. In addition, extracellular matrices (ECM) are instrumental in sustaining the localized delivery of pro-angiogenic factors (Zhang et al. 2020). The combination of pro-angiogenic biochemical cues and structural support provided by ECM scaffolds is key to enhancing tissue vascularization. Microvascular grafts represent another significant advancement in enhancing the vascularization of therapeutic cell delivery sites (Citro et al. 2019; Redd et al. 2019). These grafts are engineered to mimic the structure and function of blood vessels, providing a pre-formed vascular network when implanted into the subcutaneous space. This strategy can drastically improve the immediate availability of oxygen and nutrients to the transplanted cells, enhancing their survival and integration into the host tissue.

Mesenchymal stem cells (MSCs) play a crucial role in neo-angiogenesis, vascular regeneration, and tissue remodeling through their paracrine secretion of pro-angiogenic and trophic factors such as VEGF and basic FGF, and Transforming Growth Factor-beta (TGF-β) (Caplan and Dennis 2006). These factors directly stimulate the proliferation and migration of endothelial cells, which are essential for new blood vessel formation. Further, MSCs exert immunomodulatory functions, creating an environment conducive to angiogenesis by modulating local immune responses. This involves the downregulation of pro-inflammatory cytokines and upregulation of anti-inflammatory cytokines, which facilitates the healing and regenerative processes (Song et al. 2020). Clinically, MSCs are investigated for their potential to treat various ischemic conditions, including ischemic heart diseases, ischemic retinopathy, and peripheral/critical limb ischemia, as well as wound healing applications for burns or diabetic foot ulcers (Ankrum and Karp 2010; Cho et al. 2018; Squillaro et al. 2016). To improve MSC viability and prevent dispersion, which could attenuate their function in these therapeutic contexts, they can be embedded within scaffolds that mimic the natural extracellular matrix. The scaffold must have appropriate stability, porosity, and mechanical properties to support MSCs and facilitate their interaction with other cells and extracellular matrix components. A supportive microenvironment influences MSC differentiation and function, thereby enhancing their therapeutic efficacy (Zhao et al. 2021).

To this end, we developed a three-dimensional (3D) biodegradable scaffold using bioprinting technology. This bioprinted scaffold, comprised of MSCs embedded within an alginate and gelatin hydrogel (Alg/Gel). We characterized the Alg/Gel scaffold using rheometric and surface analyses, degradation studies, and cell viability assays *in vitro*. We assessed the efficacy of subcutaneously implanted Alg/Gel scaffold in rats and evaluated tissue development and vascularization through histological quantification of blood vessels and collagen. In summary, the bioprinted MSCs-embedded Alg/Gel scaffold when applied in a subcutaneous site, can promote localized tissue remodeling and vascularization, creating a conducive microenvironment for cellular therapy administration.

## Material and methods

### 3D printing cartridge fabrication

A custom cartridge was designed using SolidWorks software (SolidWorks 3D CAD v2023, Dassault Systèmes) to be integrated into the 3D printer 3D-Bioplotter (EnvisionTEC). The design incorporates two internal 2 ml reservoirs connected by a tubing system that leads to two coaxially aligned needles. This configuration enables the simultaneous extrusion and the homogeneous mixing of two distinct solutions at a consistent rate. The cartridge was produced using stereolithography (SLA) 3D printing technology (Formlabs, Form 3B) using clear resin (Formlabs).

### Hydrogel preparation

Alginate (Alg, Sigma), type A gelatin (Gel, Sigma), and calcium chloride (CaCl_2_, Sigma) powders were sterilized using ultraviolet (UV) irradiation for one hour. Post-sterilization, a 5% w/v alginate solution was prepared by dissolving alginate in MSC culture medium (StemXVivo) and was stirred for one hour at 37 °C. Similarly, a 15% w/v solution of type A gelatin was prepared in phosphate-buffered saline (PBS, Avantor) and agitated for one hour at 60 °C. Additionally, two distinct CaCl_2_ solutions were formulated: one at 1% w/v and another at 0.5% w/v, both dissolved in MSC culture medium and agitated for 5 min at room temperature. Following the preparation of individual solutions, the Alginate and gelatin (Alg/Gel) bio-ink was synthesized by mixing the 15% w/v gelatin solution with the 5% w/v alginate solution in a 1:3 ratio. Mesenchymal stem cells (MSCs, Cyagen), isolated from the bone marrow of F344 rats, were integrated at a concentration of 8.7 × 10^6 cells/mL in the Alg/Gel bio-ink.

### Scaffold fabrication

The scaffold was fabricated utilizing the custom double reservoir cartridge installed in the 3D bioprinter 3D-Bioplotter (EnvisionTEC). The two reservoirs of the cartridge were individually loaded with the Alg/Gel bio-ink and the 0.5% w/v CaCl_2_ solution. During the 3D printing process, the cartridge temperature was maintained at 20 °C, while the platform was pre-cooled to a temperature of 4 °C. The scaffold was printed in a 3D serpentine pattern comprising two layers with a speed of 5.5 mm/sec and pressure of 1.9 bar (Sawyer et al. 2023). Following the printing process, the scaffold was immersed in 1% w/v CaCl_2_ solution to control the level of crosslinking within the scaffold, specifically for time intervals of 5 min, 4 h, and 24 h.

### Subcutaneous implant

The subcutaneous implant structure was designed using Solidworks software (SolidWorks 3D CAD v2023, Dassault Systèmes) as previously described (Paez-Mayorga et al. 2022). It was fabricated by selective laser sintering (SLS) 3D printing (Sculpteo) using biocompatible polyamide (PA 2200, Electro Optical Systems). The top and bottom surfaces of the subcutaneous implant are composed of 2 nylon meshes, an inner nylon mesh with 300 μm × 300 μm openings, and an outer nylon mesh with 100 μm × 100 μm openings (Elko Filtering). The bottom meshes were affixed to the device using an implant-grade, biocompatible, fast-curing silicone adhesive (MED3-4213, Nusil). Before scaffold insertion within the implant, the devices underwent sterilization with ethylene oxide. Following the preparation and insertion of the scaffold, the top meshes were welded onto the device by locally melting the meshes and the device structure together. This was accomplished using a benchtop heat sealer (Weller) set at 170 °C, inside a clean laminar flow hood.

### Scanning electron microscopy (SEM) imaging

Alg/Gel scaffolds underwent a progressive ethanol dehydration process, involving immersion in ethanol solutions of increasing concentrations (30% v/v, 50% v/v, 70% v/v, 90% v/v, 100% v/v), with each immersion step lasting 2 h. Subsequently, a 2-h treatment in a critical point dryer utilizing liquid CO_2_ was performed. The scaffolds were then sputtered with a 7 nm iridium layer and imaged using the Nova NanoSEM 230 (Thermo Fisher). Imaging took place under high vacuum settings using a 5 kV electron beam at the Houston Methodist Research Institute Scanning Electron Microscopy and Atomic Force Microscopy Core.

### Atomic force microscopy (AFM)

Similarly, Alg/Gel scaffolds underwent a progressive ethanol dehydration process, following the same immersion protocol. Afterward, a 2-h treatment in a critical point dryer utilizing liquid CO_2_ was conducted. Surface roughness measurements were obtained using AFM Biocatalyst (Bruker Nano) at the Houston Methodist Research Institute Scanning Electron Microscopy and Atomic Force Microscopy Core. The AFM images were acquired in contact scanning mode using the MLCT cantilever (Bruker Nano) with a spring constant of 0.6 N/m. The images were analyzed with the software NanoScope Analysis. The image surface area difference was considered for comparing different crosslinking times. Unlike traditional roughness parameters such as average roughness and root mean square roughness, the image surface area difference provides detailed information about peaks and valleys frequency and distribution.

### Rheological tests

The rheological properties of the Alg/Gel scaffolds were assessed using an Anton Paar MCR 302e Rheometer, equipped with parallel plates of 25 mm in diameter and a gap of 1 mm. Initially, a strain sweep was conducted for each group to determine the linear viscoelastic region (LVR). Following this, a dynamic frequency sweep was carried out at a fixed strain of 0.1%—within the LVR for all Alg/Gel groups—across an angular frequency range of 0.1 to 100 Hz, utilizing a logarithmic ramp. All measurements were executed at a consistent temperature of 37 °C.

### Compression test

Alg/Gel scaffolds were subjected to compression tests on the UniVert Mechanical Test System. Utilizing a 1 N load cell (Univert), the tests were performed up to 90% of the scaffold's stretch magnitude, over a period of 180 s. Compressive strength was determined by measuring the stress at which the strain exceeded 65%, this threshold indicates the failure point of the hydrogel.

### Degradation test

To assess degradation, specific time intervals were chosen: 24 h, 48 h, 1 week, 2 weeks, 4 weeks, 5 weeks, and 6 weeks. Samples were positioned in pre-weighed cuvettes filled with 2 mL of Simulated Body Fluid (SBF) (Marques et al. 2011). Following the designated degradation period, the fluid was removed. Subsequently, the samples were frozen at -80 °C for 3 h and then transferred to a freeze dryer (Labconco™ FreeZone™ Triad Freeze Dryer) for 24 h at -40 °C under a pressure of 0.1 Torr. Post-lyophilization, the samples were weighed again to determine the extent of degradation.

### CaCl_2_ toxicity assessment

WST-1 assay (Sigma) was employed to assess the effect of calcium on cell proliferation, following manufacturer’s instructions. Cells were seeded at densities of 0.1, 0.25, 0.5, and 1 × 10^4^ cells per well in a 96-well plate and then incubated in growth medium supplemented with 1% CaCl_2_. Absorbance readings were taken at 620 nm and 450 nm, after 5 min and 24 h. Final net absorbance was calculated by subtracting the values at 450 nm from those obtained at 620 nm.

### Alg/Gel and 3D printing viability assessment

Cell viability was evaluated using the Live/Dead assay (Invitrogen) to compare the viability of MSCs after 3D printing and MSCs encapsulated in a 20% Pluronic F-127 (Sigma) hydrogel prepared with 10% MSC culture medium (StemXVivo). Cells were stained with Calcein AM and Ethidum Homodimer-1 and viability was assessed using confocal microscopy (Olympus FV3000) at 1 h and 1 day post-printing. Additionally, MSCs survival within the scaffold was further assessed at 1, 3, and 7 days post-printing and compared to MSCs in culture medium (StemXVivo).

### Efficacy study in rats

The *in vivo* efficacy study utilized eight Fisher 344 male rats (Charles River), with four animals per each group (n = 4). Each rat received sterile devices implanted subcutaneously on their dorsum. In the experimental groups, each rat was implanted with two devices: one containing an Alg/Gel scaffold embedded with 333,000 (~ 300 K) MSCs and the other containing a scaffold without MSCs. Conversely, the control group received a single device containing 500,000 (500 K) MSCs embedded in 20% Pluronic. At the conclusion of the study, four weeks post-implantation, the rats were euthanized, and the devices were explanted for analysis.

### Histopathology and Immunohistochemical (IHC) Staining

Subcutaneous implants were fixed in 10% formalin for 48 h and processed for histology. Fixed tissues were dehydrated using standard ethanol and xylene washes followed by embedding with paraffin. Sections of 5 μm were cut and stained with standard technique for hematoxylin–eosin (H&E) and Masson’s Trichrome. For blood vessel analysis, sections were stained with *Bandeiraea simplicifolia* lectin. Stained sections were visualized using a Keyence BZ-X810 inverted phase contrast microscope.

### Vascularization quantification

To quantify blood vessel density, lectin-stained images were analyzed. Ten fields of view (magnification 40x) per implant were randomly imaged and analyzed by a scientist blinded to the groups using the software QuPath. The same images were also analyzed using a convolutional neural network (CNN) imaging algorithm (Fig. S1). The CNN was trained on approximately 600 lectin-stained H&E images, with 70% used for training and 30% dedicated to validating the algorithm's performance. Vessel density was reported as the number of vessels per mm^2^ using the following equation:

$$Blood\; vessel\; density = \frac{Vessel\; number}{FOV\; area}$$

To account for the dimensions of the blood vessel, their counts were also expressed as the percentage of the total area covered by blood vessels within the field of view (FOV) area. This was calculated using the following equation:

$$Blood\; vessel\; area\; \% = \frac{Vessel\; area}{FOV\; area} \times 100$$

### Fibrotic capsule and collagen quantification

To quantify fibrotic capsule thickness and collagen area density, Masson’s Trichrome stained images were analyzed. For fibrotic capsule quantification around the Alg/Gel scaffold at least twenty fields of view (magnification 20x) were randomly imaged. Fibrotic capsule thickness was measured by a scientist blinded to the groups using the software ImageJ. For collagen quantification at least twenty fields of view (magnification 20x) per implant were randomly imaged around the Alg/Gel scaffold and the Pluronic hydrogel. Collagen area was measured through color deconvolution followed by color thresholding using a custom QuPath script by a scientist blinded to the groups. Collagen density was calculated according to the formula below.

$$Collagen\; density = \frac{Collagen\; positive\; area}{FOV\; area}$$

### Statistical analysis

All results are expressed as mean ± standard deviation. Statistical analyses were performed using Prism 9 software (GraphPad Software Inc). Two-way analysis of variance (ANOVA) with multiple comparisons test was performed to determine statistical significance of differences among groups, and p-values less than 0.05 were considered significant. Significance was indicated as follows: *p ≤ 0.05; **p ≤ 0.01; and ***p ≤ 0.001.

## Results and discussion

### Alg/Gel scaffold preparation

The Alg/Gel scaffold is composed of alginate and gelatin. Alginate, a natural polymer, is biodegradable and biocompatible, rendering it ideal for *in vivo* applications. Moreover, its viscosity is optimal for achieving a bio-ink suitable for 3D bioprinting (Axpe and Oyen 2016). Gelatin, a biopolymer derived from collagen, was incorporated in the bio-ink to improve bio-adhesivity (Łabowska et al. 2021). To enhance mechanical strength of the bio-ink, the Alg/Gel bio-ink was cross linked using divalent calcium ions (Ca^2+^) (Lee and Mooney 2012).

**Fig. 1:**
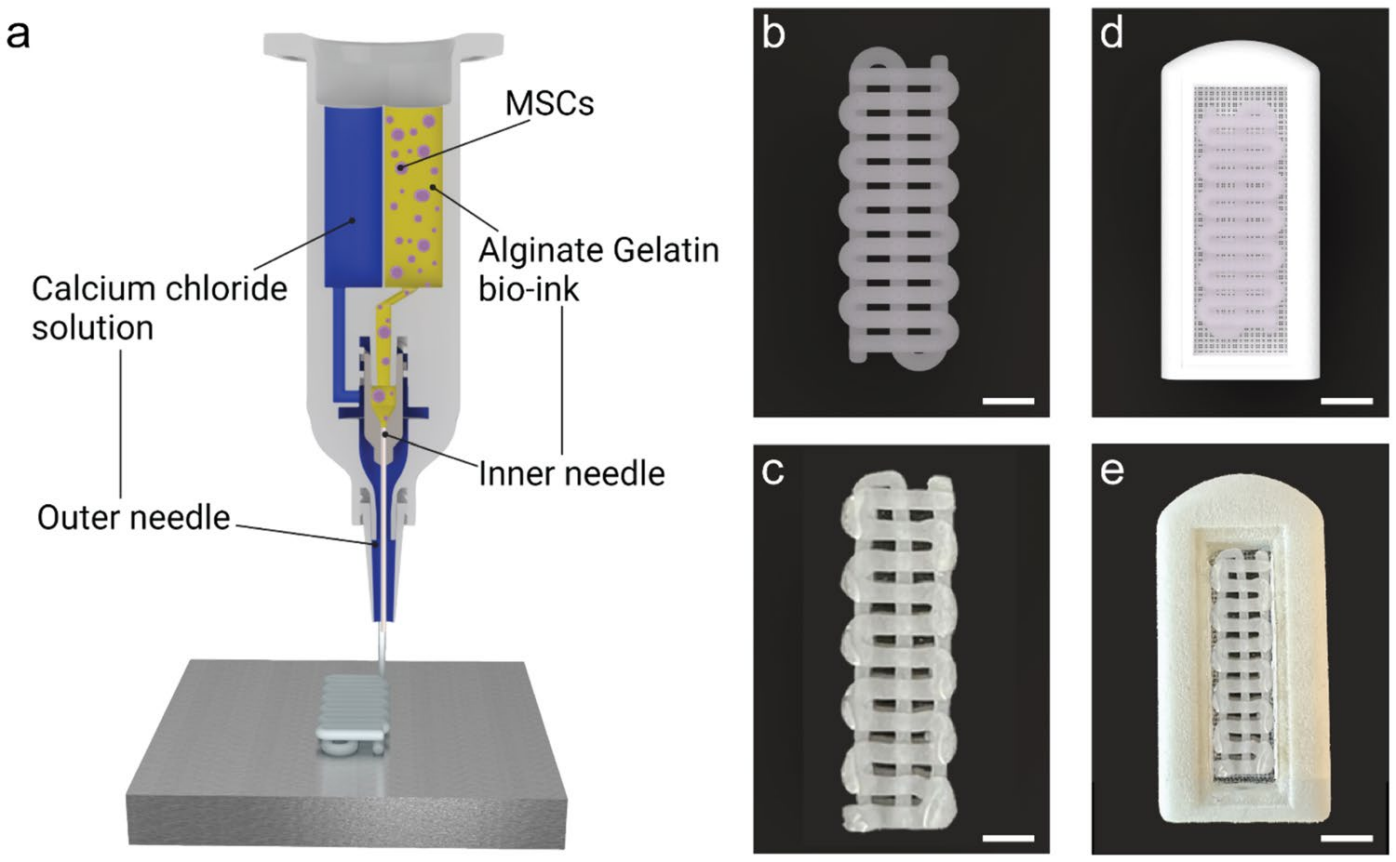
Representation of the fabrication process of the Alg/Gel scaffold. **a** Schematic of the dual reservoir cartridge used to 3D bioprint the Alg/Gel scaffold embedded with MSCs. **b** Rendering and **c** optical image of the 3D structure of the Alg/Gel scaffold (scale bars is 2.5 mm). **d** Rendering and **e** optical image of the Alg/Gel scaffold within a subcutaneous implant (scale bars is 5 mm)

The Alg/Gel scaffold was produced using a 3D bioprinting technique with a custom-made cartridge specifically designed to optimize the printability of the bio-ink (Fig. 1a). The cartridge houses two reservoirs for the Alg/Gel bio-ink and CaCl_2_ crosslinking solution, respectively. The coaxial needle design and custom printing parameters allow for the uniform extrusion of the viscous Alg/Gel bio-ink from the central needle, as the CaCl_2_ solution is simultaneously dispensed from the outer needle. This setup ensures that crosslinking occurs only upon contact with the build platform. Post-printing, the scaffold is immersed in a CaCl_2_ solution to adjust the degree of crosslinking. The Alg/Gel scaffold can be loaded with cells, such as MSCs, as demonstrated in this study, creating a bioactive scaffold (Paez-Mayorga et al. 2020a).

The Alg/Gel scaffold was designed with a serpentine structure, where intersecting filaments ensured the structural stability of the scaffold while leaving open macropores that facilitate tissue integration (Fig. 1b). The filaments were 3D bioprinted with a diameter of 1 mm, the smallest dimension that ensured printability and reproducibility of the scaffold with our setup (Fig. 1c) (Naghieh and Chen 2021). The dimensions of the scaffold (23 mm × 6 mm) can be tailored to maximize space usage within an implant (Fig. 1d). In our study, the Alg/Gel scaffold was inserted into a subcutaneous implant, providing a controlled environment for tissue growth (Fig. 1e). Additionally, a subcutaneous implant facilitates the retrieval and assessment of host tissue integration and vessel penetration.

### Alg/Gel scaffold biophysical characterization

**Fig. 2:**
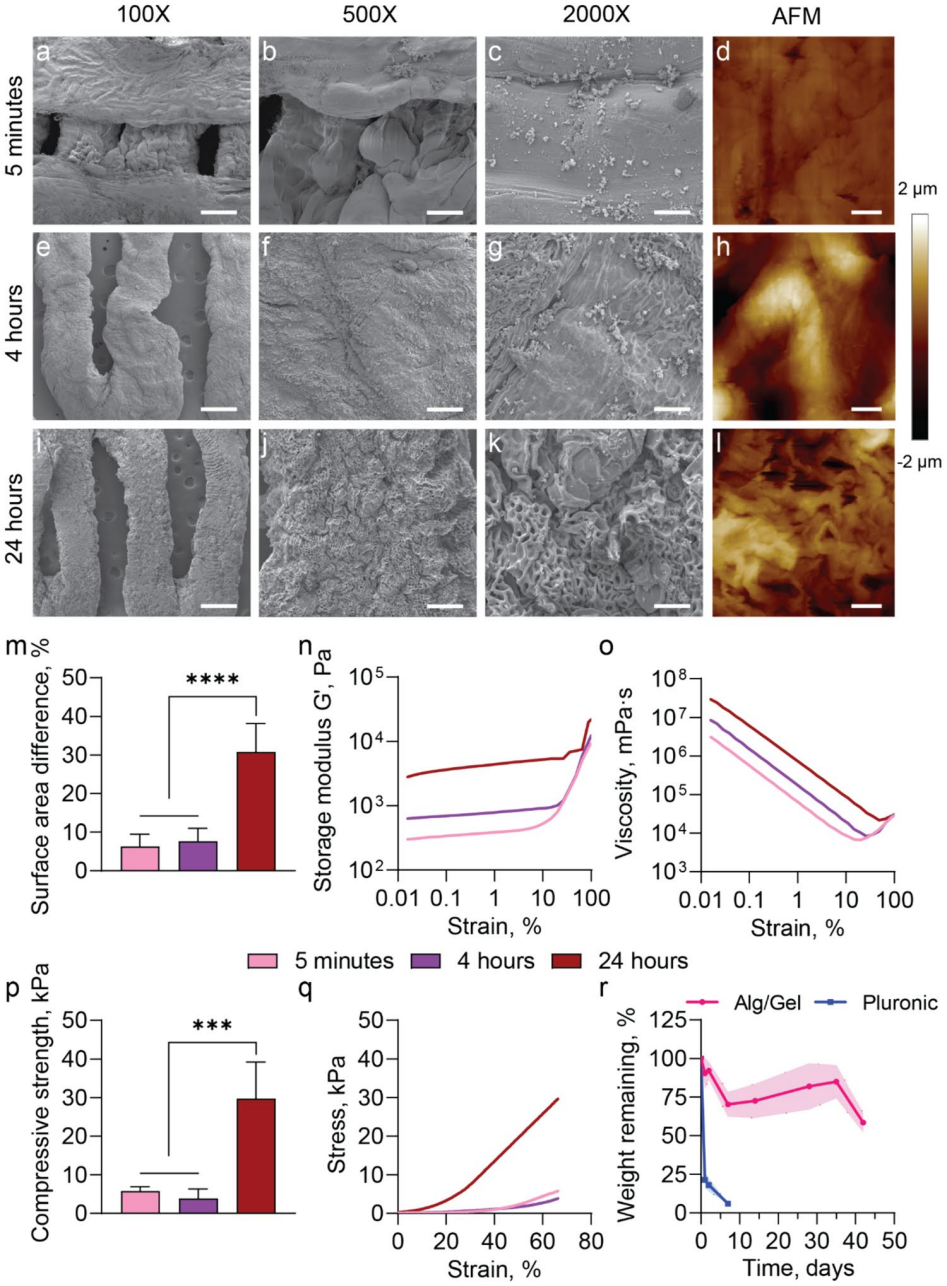
Alg/Gel Scaffold biophysical characterization. SEM image of Alg/Gel scaffold crosslinked for 5 min in 1% w/v CaCl_2_ solution **a** at 100X magnification (scale bar is 500 μm), **b** at 500X magnification (scale bar is 100 µm) and **c** at 2000X magnification (scale bar is 25 µm). **d** AFM image of Alg/Gel scaffold crosslinked for 5 min (scale bar 5 μm). SEM image of Alg/Gel scaffold crosslinked for 4 h in 1% w/v CaCl_2_ solution **e** at 100X magnification (scale bar is 500 μm), **f** at 500X magnification (scale bar is 100 µm) and **g** at 2000X magnification (scale bar is 25 µm). **h** AFM image of Alg/Gel scaffold crosslinked for 4 h (scale bar 5 μm). SEM image of Alg/Gel scaffold crosslinked for 24 h in 1% w/v CaCl_2_ solution **i** at 100X magnification (scale bar is 500 μm), **j** at 500X magnification (scale bar is 100 µm) and **k** at 2000X magnification (scale bar is 25 µm). **l** AFM image of Alg/Gel scaffold crosslinked for 24 h (scale bar 5 μm). **m** AFM roughness quantification by surface area difference percentage. Rheology tests of Alg/Gel scaffold with different crosslinking times. **n** storage modulus G’ and **o** viscosity. **p** Compressive strength and **q** stress–strain curve of compression test of Alg/Gel scaffold with different crosslinking times. **r** Degradation test of the Alg/Gel scaffold with crosslinking time of 5 min compared to Pluronic F-127 hydrogel over 6-week period

The structural and mechanical properties of Alg/Gel scaffold were assessed to select for the optimal crosslinking conditions. Crosslinking durations of 5 min, 4 and 24 h were investigated. Scanning Electron Microscopy (SEM) imaging showed that the Alg/Gel scaffolds had a homogeneous and solid macroscopic structure across all evaluated crosslinking durations (Fig. 2a, e and i). However, SEM images at the microscale revealed the formation of ripples and pores on the surface as the crosslinking time was increased. (Fig. 2b–c, f–g and j–k). These ripples, which affect the scaffold morphology, resulted from excessive ionic crosslinking. The elevated presence of inclusions, voids, or cracks on the surface of the material can act as stress concentrators, increasing its fragility. This was supported by the AFM results (Fig. 2d, h and l), where an increase in roughness was correlated with longer crosslinking time of 24 h compared to 5 min and 4 h (Fig. 2m).

Rheology test showed that the storage modulus (G’) and viscosity of the Alg/Gel scaffold increased with crosslinking duration (Fig. 2n - o). This was supported by the compression test (Fig. 2p - q), which indicated that the Alg/Gel scaffold, with a crosslinking time of 24 h, exhibited a significantly higher compressive strength than crosslinking times of 5 min and 4 h. Specifically, a significant change in mechanical properties was evident only with extended exposure to crosslinking agents (Ca^2+^ ions), in the sample treated with 24 h of crosslinking. In contrast, there was no noticeable difference in mechanical properties between the groups treated for 5 min and 4 h.

Based on these data, a short crosslinking duration of 5 min was selected, which was adequate to achieve a scaffold with sufficient structural stability. Extending the crosslinking time resulted in a more rigid but fragile scaffold, thereby increasing the likelihood of mechanical failure. Furthermore the 5-min crosslinking duration yielded mechanical properties that adequately mimic those of physiological subcutaneous tissues (Iatridis et al. 2003; Sun et al. 2021).

Next, the degradation of the Alg/Gel scaffold with crosslinking time of 5 min was compared to Pluronic F-127, which is a commonly used hydrogel for cell encapsulation and tissue engineering (Gioffredi et al. 2016; Gutowska et al. 2001; Shamma et al. 2022). Alg/Gel degradation was markedly slower, losing ~ 40% of mass during 6 weeks of incubation at 37 °C. In contrast, Pluronic rapidly degraded, losing more than 90% of the initial mass by day 7(Fig. 2r), which is consistent with previous reports (Chatterjee et al. 2019). This indicates that the Alg/Gel scaffold is still present when the process of tissue ingrowth begins, approximately four weeks post-implantation (Farina et al. 2017; Farina and Secco 2017; Paez-Mayorga et al. 2020a). This allows for the exogenous material to degrade in sync with local tissue ingrowth and remodeling.

### Effect of 3D Bioprinting on MSCs proliferation and viability

**Fig. 3:**
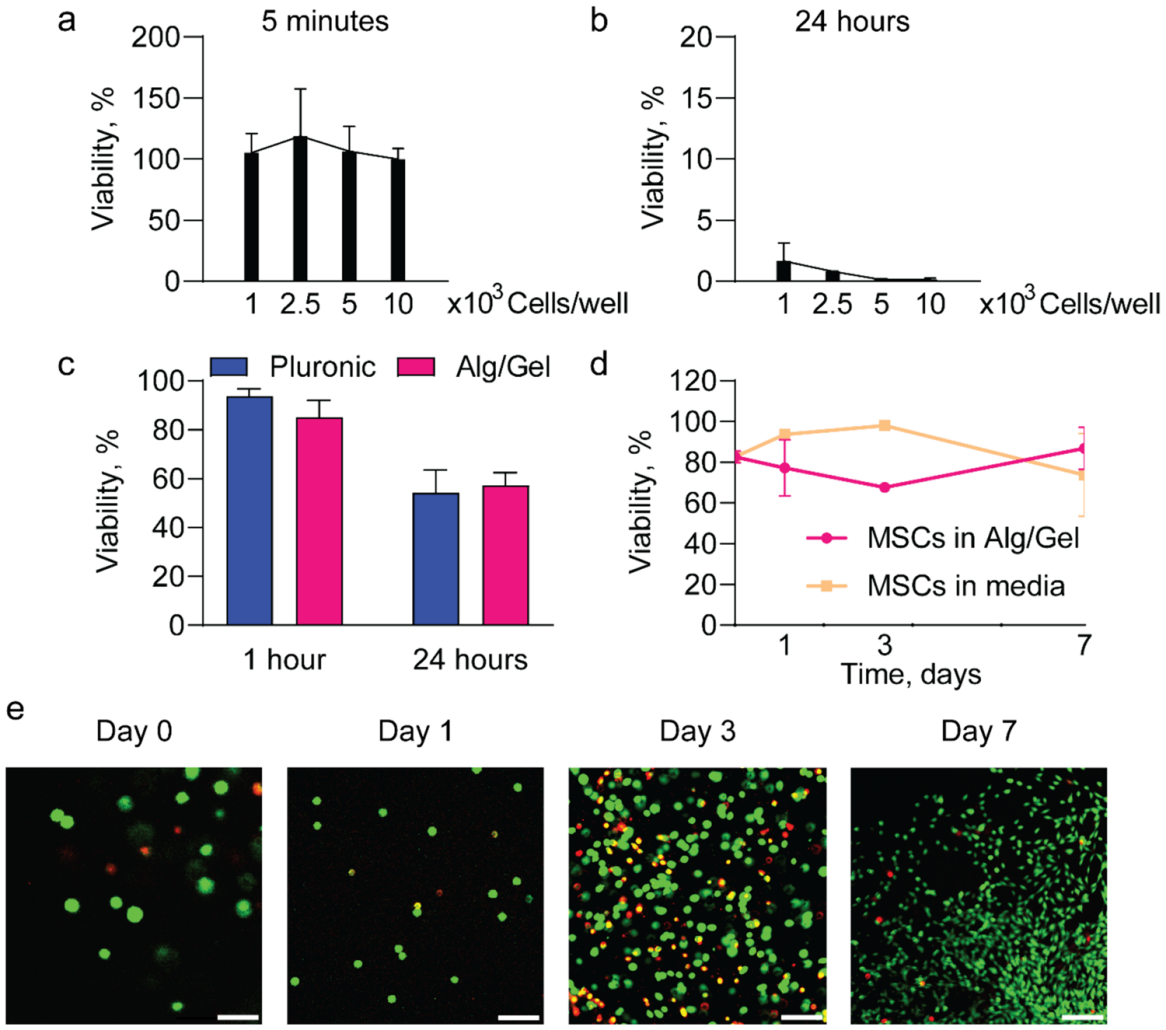
Viability of MSCs exposed to 1% CaCl₂ for **a** 5 min and **b** 24 h. **c** Comparison of MSCs viability in Alg/Gel scaffold and in Pluronic hydrogel. **d** Viability comparison between 3D bioprinted MSCs into the Alg/Gel scaffold and MSCs in culture media. **e** Images of live (green) and dead (red) MSCs 3D bioprinted after 1 h (scale bar 50 μm), 24 h (scale bar 200 μm), 3 days (scale bar 100 μm), and 7 days (scale bar 200 μm)

To assess the cytocompatibility of the scaffold, a series of cell viability assays were conducted using MSCs. A short crosslinking time of 5 min did not affect MSC viability (Fig. 3a), whereas prolonged exposure of 24 h resulted in cell death (Fig. 3b). We surmised that the high ionic strength of the solution containing Ca^2+^ ions could have induced osmotic stress, leading to cell toxicity (Cao et al. 2012). These results further support the use of a short crosslinking time of 5 min for Alg/Gel fabrication.

Next, the viability of MSCs bioprinted in Alg/Gel scaffolds was evaluated in comparison to MSCs embedded in 20% Pluronic F127 hydrogel at 1 and 24 h (Fig. 3c). No significant differences were observed in MSCs viability between the two encapsulation methods. This experiment confirmed that the 3D bioprinting process did not negatively affect cell viability compared to conventional loading methods such as that of Pluronic (Paez-Mayorga et al. 2022; Viswanath et al. 2022).

Further, MSCs viability within the bioprinted Alg/Gel was compared to MSCs cultured in media as control (Fig. 3d). MSCs in media expanded in culture, as indicated by the viability assessment on days 1 and 3. However they became over confluent by day 7, where contact inhibition affected viability. In contrast, MSCs bioprinted in the Alg/Gel scaffold showed an initial drop in viability on days 1 and 3, followed by an increase in proliferation thereafter (Fig. 3e). These data confirm that the 3D printing process does not affect the viability of cells embedded in the 3D printed Alg/Gel scaffold. Moreover, it demonstrates the capacity of the scaffold to support cell adhesion and proliferation.

### *In vivo* validation of Alg/Gel scaffold

**Fig. 4:**
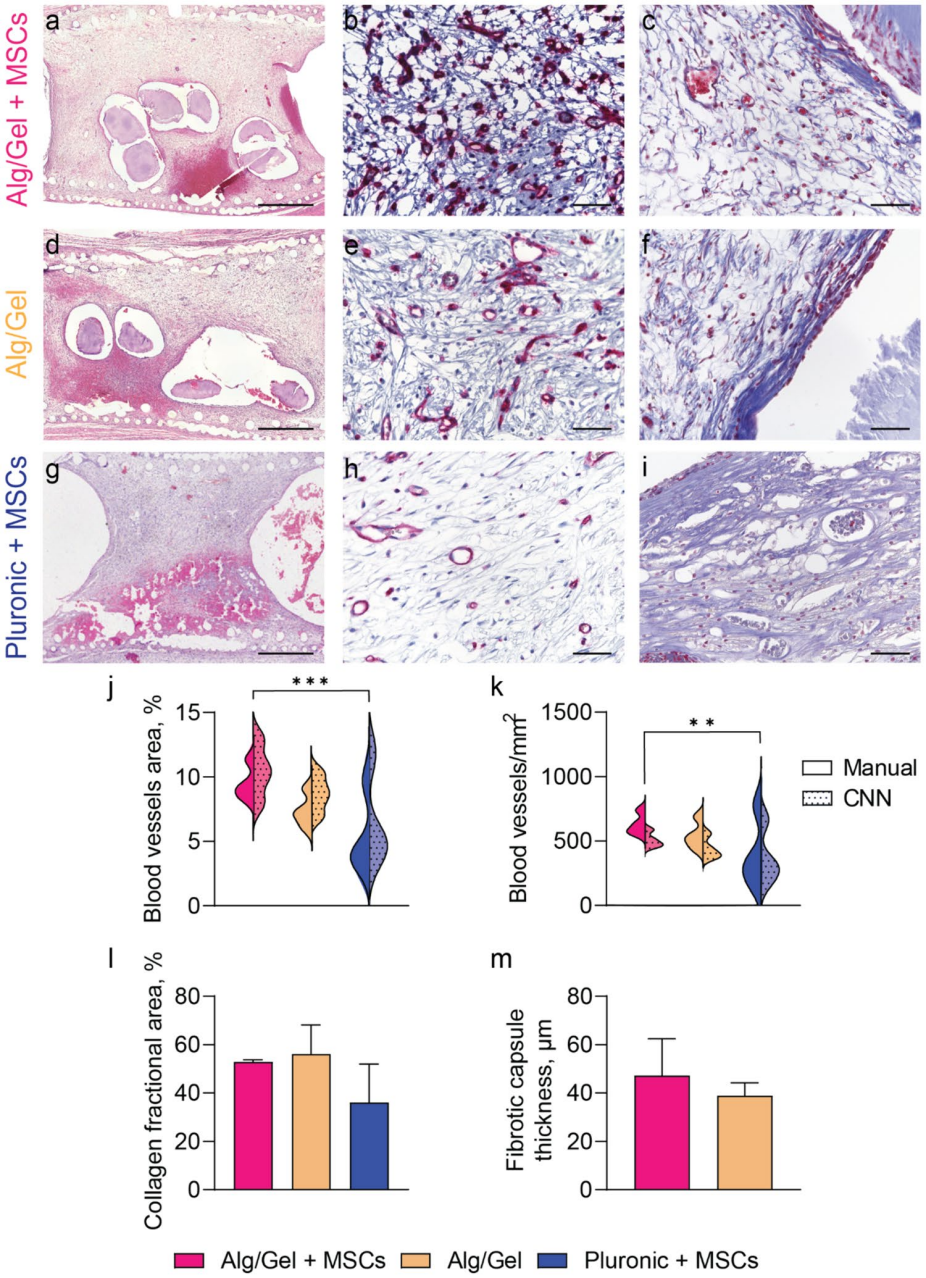
Histological images of Alg/Gel scaffold loaded with MSCs: **a** H&E (scale bar 1 mm), **b l**ectin (scale bar 50 μm) and **c** Masson’s trichrome (scale bar 50 μm) staining of the tissue inside the subcutaneous device. Histological images of Alg/Gel scaffold: **d** H&E (scale bar 1 mm), **e** lectin (scale bar 50 μm) and **f** Masson’s trichrome (scale bar 50 μm) staining of the tissue inside the subcutaneous device. Histological images of Pluronic hydrogel with MSCs: **g** H&E (scale bar 1 mm), **h l**ectin (scale bar 50 μm) and **i** Masson’s trichrome (scale bar 50 μm) staining of the tissue inside the subcutaneous device. Quantification of blood vessel **j** density and **k** area. **l** Quantification of the collagen fractional area and **m** fibrotic capsule thickness. Four samples were analyzed for each group (n = 4)

The Alg/Gel scaffold containing MSCs, Alg/Gel scaffold alone, or a Pluronic hydrogel loaded with MSCs were each individually placed in implants with openings that allow for tissue ingrowth. These implants were subcutaneously inserted in the dorsum of rats for vascularization assessment. Four weeks post-implantation, the tissues were harvested and histologically examined. Based on our previously published data, we demonstrated that a four-week timeframe was sufficient to obtain a vascularized tissue that is well integrated in the subcutaneous microenvironment (Paez-Mayorga et al. 2022, 2020a). A balance between a well-vascularized but not overly dense space can permit faster transport of oxygen and nutrients from the blood vessels to the cell graft, preventing the loss of cells due to hypoxia (Capuani et al. 2024). H&E-stained cross-section images of the ingrown tissue revealed macroscopic differences between the Alg/Gel scaffolds and Pluronic hydrogel (Fig. 4a, d and g). The Alg/Gel scaffolds showed partial degradation, in contrast to Pluronic hydrogel, which was absent, in accordance to our *in vitro* data (Fig. 2m).

Angiogenesis is crucial for the survival of transplanted cells and engineered tissues, which ensures cells receive adequate oxygen and nutrients (Gaharwar et al. 2020). Therefore, the density and dimensions of newly formed blood vessels were quantified using lectin-stained histological images (Fig. 4b, e and h). Since the manual evaluation of these features is subject to inter-observer variability, we adapted our previously developed CNN algorithm to automatically detect blood vessels on lectin-stained histological slides (Farina and Secco 2017; Zoppo et al. 2020). The CNN algorithm results confirmed the accuracy of our manual evaluation (Fig. 4j). Pluronic hydrogel loaded with ~ 500 K MSCs was previously shown to generate vascularized tissue adequate for subcutaneous cell transplantation (Paez-Mayorga et al. 2022, 2020b). In comparison, the MSC-laden Alg/Gel scaffolds induced significantly higher vascular density in the subcutaneous microenvironment than MSC-loaded Pluronic hydrogel (Fig. 4j – k), despite using a lower dose of cells (~ 300 K vs ~ 500 K, respectively). We note that Alg/Gel scaffold alone showed increase vascularization compared to the Pluronic cohort, although not statistically significant.

Additionally, Masson's Trichrome-stained histological images were used for quantification of collagen density in the newly formed tissue (Fig. 4c, f and i). Higher collagen density was observed in the tissue formed within the Alg/Gel scaffold (Fig. 4l), although not statistically significant. Further, the fibrotic capsule formed around the scaffold showed no difference in thickness between Alg/Gel scaffold with or without MSCs (Fig. 4m).

Overall, our 3D bioprinted scaffold approach could promote localized angiogenesis and tissue remodeling, which are pivotal for the long-term success of tissue engineering and cell therapeutic applications (West and Moon 2008). Furthermore, we demonstrated the potential of AI algorithms to enhance the efficiency and reproducibility of blood vessel detection.

## Conclusion

In this study, we developed a 3D bioprinted scaffold comprised of MSCs-laden Alg/Gel bio-ink to promote vascularization in a subcutaneous microenvironment. The scaffold had mechanical properties comparable to the subcutaneous tissue and supported the survival of embedded MSCs. In rats, the scaffold enhanced vascularization and tissue development in a subcutaneous environment without inducing adverse foreign body response. This approach could be advantageous for cell therapy where a supportive microenvironment is essential, such as that for the transplantation of cells with high metabolic activity, e.g., pancreatic islets, in subcutaneous spaces.

## Supplementary Information

Below is the link to the electronic supplementary material.

Supplementary file1 (DOCX 414 KB)

## Data Availability

The data used to support the findings of this study are available from the corresponding author upon request.
