# Supplementary material for: 3D bioprinted mesenchymal stem cell laden scaffold enhances subcutaneous vascularization for delivery of cell therapy
Source: Biomed Microdevices. 2024 Jun 18;26(3):29. doi: 10.1007/s10544-024-00713-2 (PMC11189315; doi:10.1007/s10544-024-00713-2)
Supplement: Supplementary file 1 — Supplementary file1 (DOCX 414 KB) [file 10544_2024_713_MOESM1_ESM.docx]

**Supplementary information**

The network has an architecture as shown in Figure S1 and consists of fully connected layers of computational nodes divided into three blocks (Farina and Secco 2017; Zoppo et al. 2020). As in classical CNNs, each layer of the network is composed of computational nodes that change their internal states according to the internal states of the entirety of the surrounding cells. Therefore the output of the analysis performed by each layer of the network is function of all the values of the computational cells composing that layer. These blocks of layers in the network are organized as follows:

1. The first block consists of 24 layers of a number of nodes equal to the number of pixels in the image and is intended to analyse the image by dividing it into 24 quadrants of identical size.
2. The second block has similar configuration to the first (24 layers of a number of nodes equal to the image’s pixels) and has the purpose of subdividing the 24 quadrants of the original image into an additional 24 quadrants. To analyse the original quadrants, the image is oversampled, that is, the original pixels are divided again into a larger number of pixels through normal oversampling techniques. This will allow the network to obtain more information.
3. The last block consists of a single row of 24 individual nodes, which is called a readout block. The purpose of this block is to transmit the output of the analysis back to the network as feedback during training.

The network was trained to automatically identify and segment blood vessels directly from histological slides. A training set of images was formed in which the objects were identified by manually tracing bounding boxes. Once an image is presented to the network, it is divided into 24 quadrants in a regular manner. Each pixel of a quadrant corresponds to a computational node of the corresponding preliminary network layer. For each node of the layer, the following calculation is performed:


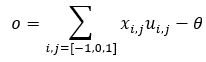


Where x corresponds to the internal computational node value of the node at position i,j, while u corresponds to the input value that can be 0 or 1 depending on whether the pixel is outside a bounding box or not. θ, on the other hand, is a threshold that can be set manually depending on the sensitivity of the system or the characteristics of the image being analysed. Next, the image goes to the second block, where each quadrant is re-subdivided into 24 sub-quadrants. If the sum of the values of or the reference sub-quadrant are above a predetermined threshold, the same calculation is performed as in the first quadrant. To distinguish the results, those obtained from the second quadrant are called o_2_. The o_2_ values for each sub-quadrant are added together and if they are above a certain threshold, the value passed to the corresponding computational node in the third block of the network will have value 1; otherwise, the value is 0. These resulting values are named o_3_ and impact on the evolution of the computational nodes’ values as follows:


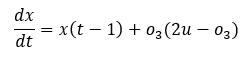


Where t represents the iteration step of the network. Once the network is trained, the value of the computational nodes is fixed. Therefore, once a new image is presented, the values o, o_2_ and o_3_ are calculated as in the training phase, determining the zones of the image where the network establishes an acceptable probability of finding a vessel. These values are printed on a digital mask, which is superimposed on the original image, presenting the output.


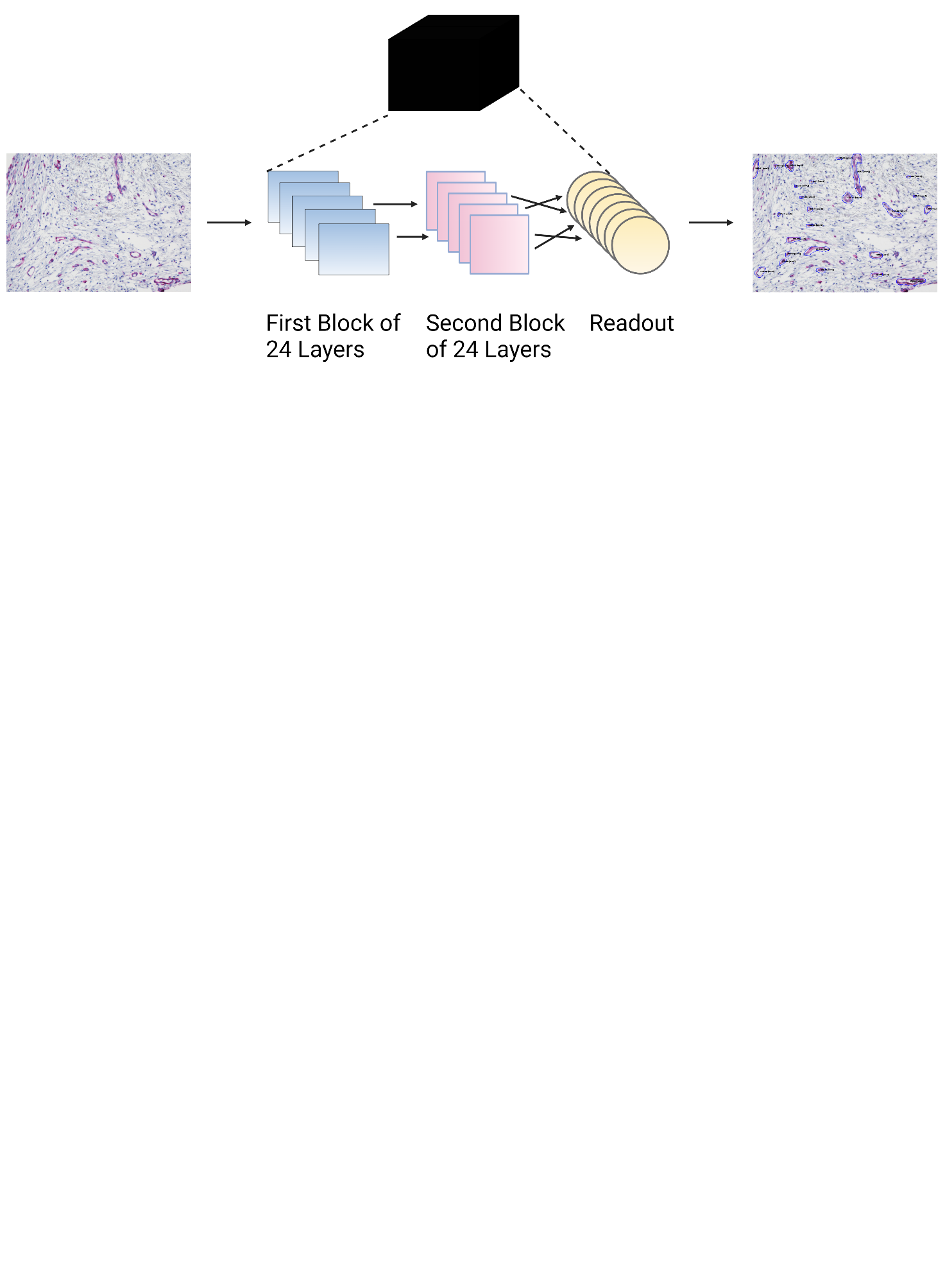


Figure S1: The CNN network used for the application comprises three blocks of 24 layers composed of computational nodes. The network was trained to automatically identify blood vessels from histological images. The three blocks analyze the image and extract the features with the highest probability of representing blood vessels. The result of the analysis is a digital mask where areas with acceptable probability are demarcated and superimposed on the original histological image to present the results.
